# Parents’ and Peers’ Autonomy Support and Exercise Intention for Adolescents: Integrating Social Factors from the Self-Determination Theory and the Theory of Planned Behaviour

**DOI:** 10.3390/ijerph17155365

**Published:** 2020-07-25

**Authors:** Alvaro Sicilia, Cornelio Águila, Magalí Posse, Manuel Alcaraz-Ibáñez

**Affiliations:** 1 Health Research Centre and Department of Education, University of Almería, 04120 Almería, Spain; asicilia@ual.es (A.S.); m.alcaraz@ual.es (M.A.-I.); 2 Department of Education, University of Almería, 04120 Almería, Spain; magaliposse@hotmail.com

**Keywords:** exercise, adolescence, autonomy support, descriptive norms, subjective norms, mediation model

## Abstract

Based on the theory of planned behaviour and self-determination theory, the objective of the present study was to analyse the relationship between (i) parents’ and peers’ autonomy support, and (ii) exercise intention in adolescents, while also considering the mediating role of attitude, control, subjective norms, and descriptive norms. A total of 428 secondary school students, aged between 13 and 19 years old (*M_age_* = 15.30, *SD* = 1.15), filled in a questionnaire assessing the variables of interest. The relationships between the study variables were examined through a mediation model with bootstrapping technique (20,000 samples) using Mplus v. 7 software. The results showed that the perception of parents’ autonomy support was positively and statistically significant associated with exercise intention; this occurring indirectly through attitude and control both in boys and girls, as well as through subjective norms in the case of girls. Conversely, the perception of peers’ autonomy support was positively and statistically significant associated with exercise intention; this occurring directly both in boys and girls, as well as indirectly through attitude in the case of girls. These findings suggest that, by involving a form of pressure (i.e., subjective/descriptive norms), perceptions of autonomy support may play a more important role than other forms of social influence in predicting exercise intention in adolescents.

## 1. Introduction

There is evidence regarding the benefits of regular exercise on an individual’s physical and mental health [1]. Despite this, it has been found that exercise practice tends to decrease throughout life, with adolescence being the period when this decrease is most pronounced [2]. Based on this evidence, it is of interest to analyse the social factors that might favour exercise intention in the adolescent population. By integrating constructs from the theory of planned behaviour (TPB) and self-determination theory (SDT), the present study examines the role of parents’ and peers’ autonomy support in exercise intention in a sample of adolescents of both genders.

### 1.1. TPB and Its Limitation in Capturing the Influence of Social Factors

TPB has often been applied to explain health-related behaviours such as exercise [3]. According to the TPB postulates, behaviour can be explained by the rational intention to engage in it, that is, the extent to which individuals consciously plan their involvement in such behaviour [3]. According to the TPB, the intention to carry out a certain behaviour (e.g., exercise) would be explained from three different constructs: the attitude towards the behaviour (i.e., the general evaluation, positive or negative, that the person makes regarding the possible results of the behaviour), the perceived control (i.e., the individual’s personal belief in their ability to control for difficulties when carrying out the behaviour), and the subjective norms (i.e., the social pressure, exerted by significant others, that the individual perceives in carrying out the behaviour) [3].

The TPB postulates have received abundant empirical support [4,5]. However, it has been observed that, in general, the capacity of attitude and control to explain intention is superior to that of subjective norms [2], which could suggest that the latter factor does not sufficiently capture the social influence posed by the TPB. One possible reason for the inferior explanatory capacity of subjective norms is that, in reflecting on what one’s significant others say should be done [3], this construct involves the existence of a source of social pressure. Therefore, although the TPB assumes that subjective norms contribute to explaining the intention to perform the behaviour, it is possible that the social pressure implicit in this construct may prevent, rather than favour, the performance of the behaviour [6].

In order to increase the explanatory capacity of the social influence exerted by the variables contemplated in the TPB, it has been suggested that descriptive norms should be incorporated into this theory [7,8]. Descriptive norms reflect the degree to which individuals perceive that their significant others are involved in the behaviour, which they are also expected to engage in. Thus, descriptive norms may have greater predictive power regarding the intention to engage in the behaviour than do subjective norms, since individuals are often guided more by what they see others doing rather than by what they are told they should do [9].

The influence of descriptive norms in adopting health-related behaviours has been previously reported. For example, the results of the meta-analysis carried out by Rivis and Sheeran [8] suggest that descriptive norms help to explain intention in an incremental way with respect to subjective norms, control, and attitude. However, evidence relating these constructs to other potentially healthy behaviours, such as exercise, has been inconclusive [9]. Specifically, the scientific literature presents evidence that supports both the absence [10] and the presence of a relationship between descriptive norms and exercise intention [11,12]. The absence of conclusive evidence invites further exploration of the relationship between these variables. This is even more necessary in adolescents, since this population is especially susceptible to peer pressure [8], a circumstance that would suggest that the role of descriptive norms might be particularly relevant at this stage of life. 

However, combining descriptive norms and subjective norms within the TPB does not avoid the restriction pointed out in the literature, namely that both reflect a social influence denoting pressure. This pressure may be explained by the fact that individuals feel pushed into conforming to the group norms, either through the messages they receive from their significant others [3] or through the behaviour model they present [8]. In this sense, an important limitation of both types of norms and, by extension, of the TPB itself, is the absence of differentiation between the different sources of social influence (e.g., parents and peers).

### 1.2. Integrating Parents’ and Peers’ Autonomy Support into the TPB

One way of complementing the TPB and understanding the influence that social factors might have on the intention to practice, is to incorporate forms of social influence that, without involving pressure, allow differences between various agents [13]. From the self-determination theory (SDT) perspective, support for autonomy is a social influence construct that reflects the degree to which individuals perceive that their significant others (i.e., parents and peers) help and encourage a certain behaviour [6]. One of the main SDT postulates is that the interpretation of the social context can favour the adoption and maintenance of the behaviour [6]. Thus, autonomous interpretations of the environment involve individuals perceiving the significant others with whom they interact (i.e., parents, peers and teachers) as favouring the possibility of choice, for example, providing reasoned explanations for carrying out the actions or recognising the subject as an actor in their own right.

Autonomy support captures a form of social influence relatively free of social pressure; hence, it reflects a social influence that is differentiated from subjective and descriptive norms [14]. For this reason, autonomy support has been proposed as a variable that might contribute to increasing the explanatory capacity of social influence measured by the TPB variables with respect to the intention to exercise [4,13]. In fact, in behaviours where initiation and maintenance involve a considerable effort (as in exercise), it would be expected that social assistance and support could be useful regardless of the control exerted and the value attributed to the activity [15]. In this regard, previous studies have suggested that autonomy support could influence the intention and practice of exercise in young people and adolescents, both directly and through the TPB variables [4,14,15]. However, these studies did not consider the role of autonomy support exercised by different agents. 

From the SDT perspective, the autonomy support construct allows one to differentiate the influence of different social agents. This contrasts with a limitation of the social factor included in the TPB; specifically, since the subjective and descriptive norms (i.e., the influence of one’s significant others) cover the interpersonal influences of those closest to the individual (e.g., family or parents), but not the influence of peer groups [16]. However, as adolescence is such an important phase for human development, peer pressure may affect the intention to exercise in a different way to that exerted by parents’ influence [17]. Therefore, integrating autonomy support into the TPB allows one to overcome this limitation and to examine the social influence of different signifiers (e.g., parents and peers).

To the best of our knowledge, there has only been one study to date [13] that has examined the influence of parents’ and peers’ autonomy support on exercise intention, taking into account the influence of the TPB variables on adolescents. Sicilia et al. [13] found that subjective norms and perceived control mediated the relationship between parents’ autonomy support and exercise intention in a sample of adolescents, while peers’ autonomy support predicted exercise intention through attitudes. Nonetheless, even though the Sicilia et al. study considered autonomy support from various agents, it did not include the influence of peer groups. By integrating autonomy support within the TPB, one can overcome this limitation and examine the social influence of different significant others (e.g., parents and peers). Furthermore, the Sicilia et al. study did not include the mediating role of descriptive norms, which could have contributed to a greater understanding of the role of social influence, as defined by different theoretical frameworks (i.e., TPB and SDT) nor did it examine possible differences in hypothesized relationships between boys and girls. A gender-differentiated analysis would be justified by the differences boys and girls show in exercise behaviour. In fact, research has repeatedly found that exercise frequency in women is lower than in men [18], this difference being especially accentuated in the adolescent stage [2]. In addition, studies examining gender differences within the TPB have shown that boys tend to show higher scores than girls in these constructs, especially in past exercise behaviour and attitudes towards exercise intention [19].

### 1.3. The Present Study

**Figure 1:**
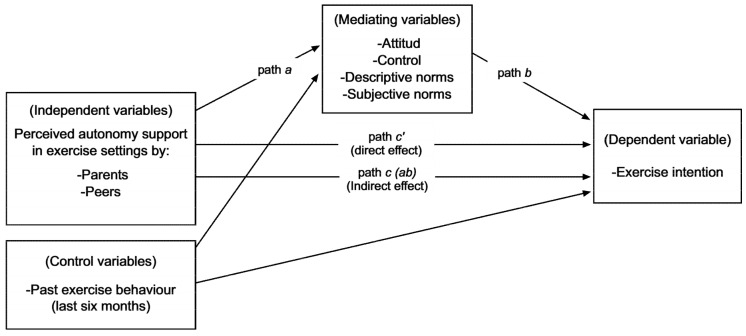
Mediation model tested.

The potential influence of autonomy support on the intention to exercise, examined through the TPB constructs, has hardly been studied. Apart from a few exceptions [13], it has been considered as referring exclusively to the influence of significant others [4,14,15]. On the other hand, research to date has not considered possible gender differences in such relationships. With these limitations in mind, the aim of this study was to examine the possible influence of autonomy support from different sources (i.e., parents and peers) on the intention to exercise in a sample of adolescents of both genders, while also considering the mediating role of descriptive norms and the other TPB constructs (i.e., attitude, perceived control, and subjective norms) (see Figure 1).

In view of the findings from previous research [13,14] it was hypothesized that parents’ and peers’ support for autonomy would predict exercise intention, both directly and mediated through the TPB variables. Although it is expected that the relationships between the variables may be affected by gender, given the exploratory nature of the sex-segmented analysis, no prior hypotheses were made.

## 2. Materials and Methods 

### 2.1. Participants

A total of 428 students in secondary and high school education (211 boys and 217 girls) participated in the study. They were aged between 13 and 19 years (*M*_age_ = 15.30, *SD* = 1.15) and attended two centres located in a Spanish provincial capital.

### 2.2. Measures 

Theory of planned behaviour variables. The items of the TPB variables (attitude, subjective norms, perceived behavioural control and exercise intention) were measured using the TPB questionnaire on exercise [20]. These items were adapted and validated in the Spanish population by González-Cutre, Sicilia, Beas-Jiménez and Hagger [21]. This questionnaire consists of 15 items that are answered using a 7-point Likert response scale. The following four variables are assessed:

Attitude: This construct was measured using a seven-point scale, where at the end of five items, 5 pairs of bipolar adjectives were placed (boring/interesting, unenjoyable–enjoyable, bad–good, useless–useful, harmful–beneficial), answering the item “participating in physical activity and sport during my leisure-time”. The Cronbach’s alpha value (α) was 0.89 for this scale in the study by González-Cutre et al. [21].

Subjective norms: These were measured using 4 items (e.g., “most people close to me expect me to do active sports and/or physical activities during my leisure time”) which were answered on a Likert-type scale from 1 (strongly agree) to 7 (strongly disagree). The α value in the study by González-Cutre et al. [21] was 0.83.

Perceived behavioural control: This was measured using 3 items (e.g., “how much control do I have over doing active sports and/or physical activities in my leisure time”) that were answered on a Likert scale from 1 (no control) to 7 (total control). The α value in the study by González-Cutre et al. [21] was 0.84.

Exercise intention: This was measured using 3 items (e.g., “I plan to do active sports and/or physical activity during my leisure time in the next 5 weeks”) that were answered on a Likert scale from 1 (strongly agree) to 7 (strongly disagree). The α value in the study by González-Cutre et al. [21] was 0.84.

Descriptive norms: A direct translation of the English version by Priebe and Spink [7] was used, responding to two questions: “How many important people for you are engaged in physical exercise?” answered on a Likert scale from 1 (nobody) to 7 (everybody), and “Think of the important people for you; what percentage are engaged in physical exercise?” answered on a Likert scale from 1 (0%) to 7 (100%).

Parents’ and peers’ perceived autonomy support: The version validated in the Spanish context [22] of the Perceived Autonomy Support Scale in Exercise Settings [23] was used, taking into account two different social factors: parents’ and peers’ autonomy support. The scale was headed by the statement “In my physical activity or sport...” and is composed of 12 items (e.g., “Parents/peers provide me with choice and options about how to do physical activity/sport during my leisure time”), grouped into two factors (parents’ and peers’ autonomy respectively), which were answered on a Likert-type scale from 1 (strongly disagree) to 7 (strongly agree). The α value was 0.96 for parents’ autonomy support and 0.95 for peer autonomy support.

Past exercise behaviour: This was measured using a single item: “In the course of the past 6 months, how often, on average, have you participated in vigorous physical activities for 20 minutes at a time?” Responses were collected on a Likert scale ranging from 1 (not at all) to 6 (most days of the week). This measure has been used in numerous previous studies to estimate past exercise behaviour in terms of frequency [4,13,14,21].

### 2.3. Procedure

In order to translate the items assessing the descriptive norms into Spanish, the reverse translation strategy of Hambleton [24] was used, seeking to make the translated version equivalent to the original at a semantic level. The items were translated into Spanish by a team of two translators. Afterwards, two further translators translated the items back to their original language. The resulting version was analysed by a group of graduates in psychology (*N* = 1) and physical activity and sport sciences (*N* = 2) [25], who did not suggest the need for modifications.

Once the final version of the items was obtained, two public high schools were contacted to request their collaboration in the study. Since the participants were minors, they were asked for written parental authorization to participate in the study. The questionnaire was administered in the presence of a member of the research group who, after reporting the anonymous nature of the study, was available to the participants to resolve any doubts that might have arisen during the data collection process. The participants needed approximately 10 minutes to complete the questionnaire.

### 2.4. Data Analysis

Firstly, the existence of outliers was considered. No cases were observed in which the standardized score for any of the variables exceeded the absolute value of 4 [26], so all cases were used in the subsequent analyses. Secondly, descriptive statistics and bivariate correlations were obtained between the study variables (Pearson’s *R*), in this case using IBM SPSS v.24 software (Armonk, NY, USA). The effect size (*d_Cohen_*) of the differences between girls and boys in these variables was then calculated using the procedure and interpretation criteria described by Cohen [27]. Thus, *d_Cohen_* values below 0.20 indicate a negligible effect, between 0.20 and 0.49 a small effect, between 0.50 and 0.79 an intermediate effect, and equal to or greater than 0.80 a large effect. The hypothesized model (see Figure 1) was then tested independently for girls and boys using a path analysis technique [28] in Mplus v.7 software [29]. In view of the results of the multivariate normality test (i.e. Mardia coefficient, *p* < 0.001), and in order to avoid overestimating the indirect effects of the model, a bootstrapping technique of 20,000 samples was applied to obtain the total direct and indirect effects involved, as well and their 95% bias corrected confidence interval (CI) [30]. CIs not comprising the zero value indicate statistically significant effects, without it being necessary for the two direct effects involved (i.e., Path a and Path b) to be statistically significant in the case of indirect effects [30]. Finally, the effect size (*q_Cohen_*) of the differences in direct and indirect effects across girls and boys was calculated using the procedure and interpretation criteria described by Cohen [27]. Thus, values of *q_Cohen_* below 0.10 indicate a negligible effect, between 0.10 and 0.29 a small effect, between 0.30 and 0.49 an intermediate effect, and equal to or greater than 0.50 a large effect. A statistical significance level of *p* < 0.05 was used in all analyses.

## 3. Results

### 3.1. Preliminary Analysis

The results shown in Table 1 reveal medium-to-high levels in all the study variables (i.e., scores above the midpoint of the respective scales). Medium-to-large-sized positive correlations were observed between all the study variables. The scores tend to favour boys, with effect sizes ranging from small (parents’ autonomy support) to medium (past exercise behaviour).

### 3.2. Main Analysis

**Figure 2:**
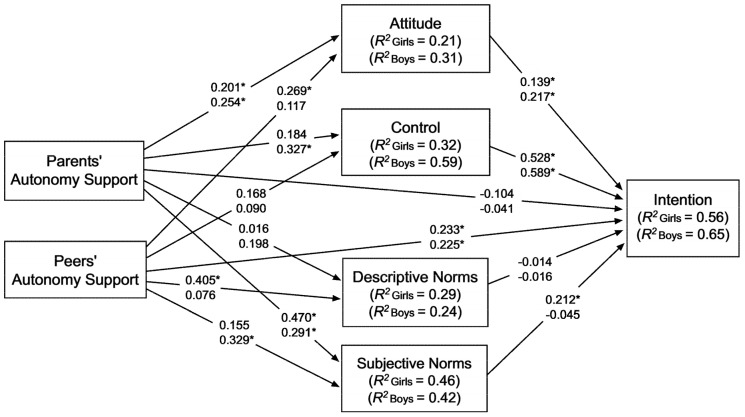
Direct effects and explained variance in the mediation model. Unstandardized regression coefficients shown were obtained by applying 20,000 bootstrapping iterations. The values in the top (bottom) row show the estimates for girls (boys). For the sake of clarity, the following values have not been included in the graphical representation: (a) the effects of the control variable (i.e., past exercise behaviour in the last six months), and (b) the covariance terms between parents’ and peers’ autonomy support. * Denotes a statistically significant regression coefficient (i.e., the 95% CI does not contain the zero value).

The results of the mediation model are shown in Figure 2 and Table 2. These results indicate that, after controlling for the effects of past exercise behaviour, perceived parents’ autonomy support was positively associated with: (i) attitude (statistically significant for girls and boys), (ii) control (statistically significant only for boys), and (iii) subjective norms (statistically significant for girls and boys). In turn, the perception of peers’ autonomy support was positively associated with (i) attitude (statistically significant for girls only), (ii) descriptive norms (statistically significant for girls only), and (iii) subjective norms (statistically significant for boys only). Attitude and control were positively and statistically significant associated with intention in both girls and boys, while subjective norms were positively and statistically significant associated with intention in girls only. 

Perceived parents’ autonomy support was positively and statistically significant associated with exercise intention, specifically through (i) attitude and control (in both girls and boys), and (ii) subjective norms (in the case of girls). Perceived autonomy support from peers was positively and statistically significant associated with exercise intention, specifically through attitude (in girls only). In addition, perceived peers’ autonomy support was positively and statistically significantly associated with exercise intention in a direct way in both girls and boys.

Past exercise behaviour over the previous six months was positively and statistically significant associated with exercise intention, both in girls (B = 0.223, CI 95% = 0.078, 0.366) and boys (B = 0.270, CI 95% = 0.128, 0.432). Overall, the considered variables explained 56% of the variance in exercise intention in the case of girls and 65% in the case of boys. 

## 4. Discussion

The objective of this study was to examine the influence of parents’ and peers’ autonomy support on exercise intention in a sample of adolescent boys and girls, considering the mediating role of descriptive norms and the other TPB constructs. The results suggest that the social factors represented by autonomy support from different agents (i.e., parents and peers) may play a more relevant role in explaining exercise intention than do the social factors coming from the TPB (i.e., subjective norms and descriptive norms). On the other hand, the influence of autonomy support on exercise intention occurred via different routes. While parents’ autonomy support was associated with exercise intention indirectly, through some of the TPB constructs, peers’ autonomy support was associated with exercise intention directly on the whole. The results of the present study help to clarify the role of social influence within the TPB.

Regarding the role of social factors represented by the TPB, the results are in line with previous studies, since they suggest that these factors might explain exercise intention to a lesser extent than the rest of the TPB constructs [10,14]. In fact, the effect of subjective norms on exercise intention was only of some relevance in the case of girls, while the effect of descriptive norms was not relevant in either of the groups. Several explanations might explain these findings. First, previous research has shown a high correlation between attitudes and subjective norms [9], suggesting that the effect of subjective norms on exercise intention may be somewhat absorbed by attitude. Second, the influence of descriptive norms on behaviour (i.e., what I perceive other signifiers already do) has been found especially in behaviours that present a health risk, such as the consumption of alcohol and other drugs [8]. However, this effect may be present less in behaviours that, far from representing a health risk, promote it instead (i.e., exercise). Finally, previous research has shown that individuals tend to be influenced by the attitudes and intentions of their groups, to the extent that they identify with them [17,31]. In this sense, a possible explanation for the limited explanatory capacity shown by the descriptive and subjective norms in explaining exercise intention might come from the lack of specificity regarding the relevance of the social reference group. In fact, at the time of measuring these norms, a general question was asked about significant others, but not about specific groups of relevance. Bearing in mind that reference groups vary between individuals and depending on the type of behaviour studied [32], this may have affected the relationship between the social norms measured within the TPB and exercise intention. 

In contrast to the limited explanatory power of the TPB social factors in explaining exercise intention, in the case of the social factors represented by the autonomy support variable, these were shown to be superior. This result may be explained by the differentiated characteristics of the social factors coming from the TPB and SDT. The TPB constructs (i.e., subjective and descriptive norms) involve a certain degree of social pressure, to the extent that they assess the degree to which individuals perceive that their significant others indicate what should be done, or serve as a model of behaviour [7]. In contrast, autonomy support assumes an interpersonal context that is relatively free of social pressure [14], since it assesses the degree to which individuals perceive possibilities of choice [6]. It is precisely this greater absence of pressure that might explain its greater predictive power on exercise intention. Indeed, previous research has shown that the effects of autonomy support on intentions, behaviour and attitudes are not strongly affected by social identity and the feeling of belonging to the group [4]. This suggests that the influence of other signifiers may further affect the intention to carry out the behaviour when this influence is perceived in the form of autonomy support, regardless of whether individuals identify with their group. Therefore, the results of the present study suggest that social factors arising from the TPB and SDT reflect different interpersonal contexts, so that including the influence of autonomy support within the TPB might contribute to an understanding of how social influences shape exercise intention [9,13]. 

The results of the present study further suggest that the effects of parents’ autonomy support on exercise intention would be different from the effects of peers’ autonomy support. Thus, while parents’ autonomy support predicted exercise intention indirectly, through subjective norms (particularly in girls), the effects of peers’ autonomy support were mainly direct. These results are partially in line with those reported by Sicilia et al. [13], particularly since the indirect effects of parents’ and peers’ autonomy support on exercise intention were produced through the TPB variables. However, it should be noted that Sicilia et al. did not examine the possible direct effects that social influence from these agents might have on intention. In any case, the differences between the direct and indirect effects observed in the present study suggest that a more complete autonomy support, such as that seemingly received from parents, would facilitate the internalization of the behaviour to a greater extent, as well as the intention to perform it in future. In fact, previous research has shown that autonomy support facilitates consistency between affective responses and intention to perform the behaviour, which reflects a greater degree of behaviour internalization, given that the intention to perform the behaviour is produced from internal psychological states (beliefs, attitudes and emotions) [6,15]. In contrast, the direct effect of peers’ autonomy support, without the mediation of attitude, might reflect an influence perceived as not being completely autonomous, so that peers’ influence might shape intention regardless of any previous unfavourable attitudes towards exercise [14].

Some of the gender differences in the relationships examined are notable. First, while the indirect effect of parents’ autonomy support on exercise intention was exerted through attitude and control in the case of boys, it was exerted through attitude and subjective norms in the case of girls. This suggests that attitude (that is, the value that an individual attributes to the future behaviour to be performed) is an essential factor in both boys and girls when it comes to assessing the degree of internalization of the activity into their own identity [15]. However, apart from the essential mediating role that attitude seems to play in both groups, the existence of a mediation effect through subjective norms in the case of girls suggests that they could be perceiving the influence coming from parents in a more autonomous way. In fact, for girls, parents’ autonomy support explained a greater variance in subjective norms than for boys, suggesting that the former may have understood parental influence as an interest in their needs and autonomy rather than as a pressure [16]. In this regard, Sas-Nowosielski [33] found that girls are more sensitive to their families’ influence on exercise than boys are. Similarly, Abraído-Lanza, Shelton, Cunha and Crookes [34], in a study with Dominican women, found that those who reported greater family ties and perceived their family members as supporting them, manifested exercise practice more often. In the case of boys, the results of the present study suggest that perceived control, along with attitudes, mediates the relationship between parents’ autonomy support and exercise intention rather than subjective norms. This result may be logical if we consider that boys not only show a higher frequency of exercise than girls, but that they are more competent in this domain [35]. As previous research has shown [13,21], a positive perception of current competence in the context of exercise is associated with possible socio-cognitive judgments that the individual may make regarding future success in that behaviour. 

On the other hand, although the effect of peers’ autonomy support on intention to exercise was mainly direct, it should be noted that there was a small attitudinal mediating effect in the case of girls. This result suggests that the influence of peers’ autonomy support may be more conducive to internalizing exercise behaviour in girls than in boys by making behaviour more dependent on the value attributed to it. As Ryan and Deci [6] have pointed out, this occurs when social influence is perceived by the individual as truly autonomous. Since behaviour is internalized in the individual’s identity, it is more likely that the intentions are in line with their affective system [15]. 

It is noteworthy that, despite the small differences indicated, the relationships in the model were quite stable in both groups, reinforcing the idea that autonomy support might play a relevant role in explaining exercise intention. The fact that parents’ and peers’ autonomy support seems to affect exercise intention through different processes suggests that integrating these constructs within the TPB may lead to a more precise explanation of the influence of social factors on this variable. 

## 5. Conclusions

This study contributes to clarify the role of social influence within the TPB. The results suggest that the SDT social factors might play a more relevant role in explaining exercise intention in adolescents than the TPB social factors. In fact, the perception of both parents’ and peers’ autonomy support have been the social factors that showed the strongest positive relationship with exercise intention. In contrast, both subjective and descriptive norms showed a weak relationship with exercise intention. Therefore, social factors that lead individuals to perceive an absence of pressure on the intention or performance of a behaviour (such as autonomy support) might favour intention to engage in exercise, while those factors involving different forms of social pressure (such as subjective and descriptive norms), might rather deter individual from engaging in future behaviour. In any case, the results of the present study suggest that considering different social factors from different agents could increase the explanatory capacity when studying the social influence on exercise intention and behaviour.

Despite the possible contribution of the results of the present study, several limitations should be highlighted. Firstly, the transversal design employed does not allow causal relationships to be established. Secondly, although the descriptive and the subjective norms within the TPB have been included in the present study, other variables that might be affecting the relationships studied (e.g., identity or group membership) have not been measured. Future studies should examine the effects of autonomy support from different agents, considering also other constructs that might affect social influence norms such as identification with the group. Finally, it should be noted that the present study measured exercise intention rather than exercise behaviour. While it is true that exercise intention is often highly correlated with behaviour [36], future studies should replicate the result of this study by also assessing exercise behaviour, directly if possible. 

## Figures and Tables

**Table 1:** Descriptive Statistics and Bivariate Correlations among Variables.

Variable	Range	α		*M (SD)*		*d_Cohen_*	Skewness		Kurtosis		1	2	3	4	5	6	7	8
		Girls	Boys	Girls	Boys		Girls	Boys	Girls	Boys								
1. Past exercise behaviour (last 6 months)	1–6	-	-	3.80 (1.43)	4.52 (1.40)	0.51	−0.01	−0.71	−0.80	−0.35	-	0.33	0.30	0.25	0.47	0.35	0.39	0.49
2. Parents’ Autonomy Support	1–7	0.96	0.95	5.19 (1.44)	5.50 (1.23)	0.23	−0.68	−0.88	−0.35	0.49	0.45	-	0.71	0.40	0.43	0.39	0.64	0.43
3. Peers’ Autonomy Support	1–7	0.96	0.94	4.98 (1.33)	5.35 (1.11)	0.30	−0.61	−0.72	−0.16	0.54	0.42	0.66	-	0.41	0.41	0.50	0.54	0.48
4. Attitude	1–7	0.90	0.95	5.43 (1.49)	5.82 (1.31)	0.28	−0.87	-1.40	−0.15	1.93	0.49	0.46	0.40	-	0.41	0.26	0.47	0.45
5. Control	1–7	0.84	0.86	5.09 (1.36)	5.50 (1.24)	0.32	−0.54	−0.69	−0.17	−0.31	0.69	0.61	0.51	0.51	-	0.34	0.49	0.66
6. Descriptive Norms	1–7	0.80	0.80	4.59 (1.27)	4.92 (1.24)	0.26	−0.27	−0.52	−0.19	−0.02	0.44	0.39	0.33	0.35	0.48	-	0.40	0.35
7. Subjective Norms	1–7	0.89	0.85	5.21 (1.42)	5.52 (1.16)	0.24	−0.62	−0.67	−0.31	−0.17	0.41	0.58	0.58	0.49	0.58	0.43	-	0.54
8. Exercise Intention	1–7	0.97	0.95	4.91 (1.74)	5.52 (1.59)	0.37	−0.35	−0.88	-1.05	−0.16	0.68	0.52	0.52	0.56	0.75	0.40	0.49.	-

Note. The correlation values shown above the diagonal correspond to girls (*n* = 217). The correlation values shown below the diagonal correspond to boys (*n* = 211). All correlation values shown are statistically significant (*p* < 0.001). α = Cronbach’s alpha.

**Table 2:** Direct and Indirect Effects of the Tested Mediation Model.

Sex	Path *a* (IV→MV)					Path *b* (MV→IV)					Path *c’* (IV→DV)					Path *c* (IV→MV→DV)				
	Variables	*B*	95% CI		*q_Cohen_*	Variables	*B*	95% CI		*q_Cohen_*	Variables	*B*	95% CI		*q_Cohen_*	Variables	*B*	95% CI		*q_Cohen_*
			Low	Up				Low	Up				Low	Up				Low	Up	
Girls	PaAS→Att	0.201 *	0.015	0.377	0.046	Att→Int	0.139 *	0.007	0.282	0.062	PaAS→Int	−0.104	−0.280	0.086	0.055	PaAS→Att→Int	0.028 *	0.002	0.081	0.002
Boys	PaAS→Att	0.254 *	0.095	0.420		Att→Int	0.217 *	0.060	0.409		PaAS→Int	−0.041	−0.195	0.129		PaAS→Att→Int	0.055 *	0.015	0.124	
Girls	PaAS→Con	0.184	0.000	0.382	0.135	Con→Int	0.528 *	0.367	0.692	0.058	PaAS→Int	−0.104	−0.280	0.086	0.055	PaAS→Con→Int	0.097 *	0.003	0.208	0.070
Boys	PaAS→Con	0.327 *	0.165	0.490		Con→Int	0.589 *	0.382	0.800		PaAS→Int	−0.041	−0.195	0.129		PaAS→Con→Int	0.193 *	0.089	0.350	
Girls	PaAS→DN	0.016	−0.144	0.180	0.182	DesN→Int	−0.014	−0.187	0.145	0.003	PaAS→Int	−0.104	−0.280	0.086	0.055	PaAS→DesN→Int	0.000	−0.020	0.012	0.002
Boys	PaAS→DN	0.198	−0.001	0.371		DesN→Int	−0.016	−0.137	0.099		PaAS→Int	−0.041	−0.195	0.129		PaAS→DesN→Int	−0.003	−0.036	0.020	
Girls	PaAS→SN	0.470 *	0.285	0.656	0.201	SubN→Int	0.212 *	0.028	0.396	0.208	PaAS→Int	−0.104	−0.280	0.086	0.055	PaAS→SubN→Int	0.100 *	0.017	0.215	0.084
Boys	PaAS→SN	0.291 *	0.115	0.461		SubN→Int	−0.045	−0.188	0.113		PaAS→Int	−0.041	−0.195	0.129		PaAS→SubN→Int	−0.013	−0.068	0.030	
Girls	PeAS→Att	0.269 *	0.082	0.447	0.145	Att→Int	0.139 *	0.007	0.282	0.062	PeAS→Int	0.233 *	0.020	0.429	0.023	PeAS→Att→Int	0.037 *	0.003	0.103	0.011
Boys	PeAS→Att	0.117	−0.068	0.301		Att→Int	0.217 *	0.060	0.409		PeAS→Int	0.225 *	0.051	0.402		PeAS→Att→Int	0.025	−0.009	0.103	
Girls	PeAS→Con	0.168	−0.033	0.370	0.080	Con→Int	0.528 *	0.367	0.692	0.058	PeAS→Int	0.233 *	0.020	0.429	0.023	PeAS→Con→Int	0.089	−0.014	0.225	0.031
Boys	PeAS→Con	0.090	−0.055	0.237		Con→Int	0.589 *	0.382	0.800		PeAS→Int	0.225 *	0.051	0.402		PeAS→Con→Int	0.053	−0.029	0.159	
Girls	PeAS→DN	0.405 *	0.233	0.573	0.385	DesN→Int	−0.014	−0.187	0.145	0.003	PeAS→Int	0.233 *	0.020	0.429	0.023	PeAS→DesN→Int	−0.006	−0.085	0.057	0.003
Boys	PeAS→DN	0.076	−0.103	0.245		DesN→Int	−0.016	−0.137	0.099		PeAS→Int	0.225 *	0.051	0.402		PeAS→DesN→Int	−0.001	−0.027	0.008	
Girls	PeAS→SN	0.155	−0.034	0.354	0.177	SubN→Int	0.212 *	0.028	0.396	0.208	PeAS→Int	0.233 *	0.020	0.429	0.023	PeAS→SubN→Int	0.033	−0.002	0.115	0.026
Boys	PeAS→SN	0.329 *	0.172	0.505		SubN→Int	−0.045	−0.188	0.113		PeAS→Int	0.225 *	0.051	0.402		PeAS→SubN→Int	−0.015	−0.067	0.036	

Note. IV = independent variable; MV = mediating variable; DV = dependent variable; CI = confidence intervals; Low = lower bound; Up = upper bound; PaAS = parents’ autonomy support; PeAS = peers’ autonomy support; DesN = descriptive norms; SubN = subjective norms; Att = attitude; Con = control; Int = intention. Unstandardized regression coefficients (B) and confidence intervals (CI) were obtained by applying 20,000 bootstrapping iterations. * Denotes a statistically significant regression coefficient (i.e., the 95% CI does not contain the zero value).
